# Massively parallel electro-optic sampling of space-encoded optical pulses for ultrafast multi-dimensional imaging

**DOI:** 10.1038/s41377-023-01077-7

**Published:** 2023-02-15

**Authors:** Yongjin Na, Hyunsoo Kwak, Changmin Ahn, Seung Eon Lee, Woojin Lee, Chu-Shik Kang, Jungchul Lee, Junho Suh, Hongki Yoo, Jungwon Kim

**Affiliations:** 1 Korea Advanced Institute of Science and Technology (KAIST), Daejeon, 34141 Korea; 2 Korea Research Institute of Standards and Science (KRISS), Daejeon, 34113 Korea

**Keywords:** Frequency combs, Optical metrology, Imaging and sensing, Microwave photonics, Ultrafast photonics

## Abstract

High-speed and high-resolution imaging of surface profiles is critical for the investigation of various structures and mechanical dynamics of micro- and nano-scale devices. In particular, recent emergence of various nonlinear, transient and complex mechanical dynamics, such as anharmonic vibrations in mechanical resonators, has necessitated real-time surface deformation imaging with higher axial and lateral resolutions, speed, and dynamic range. However, real-time capturing of fast and complex mechanical dynamics has been challenging, and direct time-domain imaging of displacements and mechanical motions has been a missing element in studying full-field structural and dynamic behaviours. Here, by exploiting the electro-optic sampling with a frequency comb, we demonstrate a line-scan time-of-flight (TOF) camera that can simultaneously measure the TOF changes of more than 1000 spatial coordinates with hundreds megapixels/s pixel-rate and sub-nanometre axial resolution over several millimetres field-of-view. This unique combination of performances enables fast and precise imaging of both complex structures and dynamics in three-dimensional devices and mechanical resonators.

## Abstract (web summary)

Ultrafast multidimensional imaging is made possible by the electro-optic sampling of arrival timings of multiple space-to-wavelength encoded light pulses.

## Graphical abstract

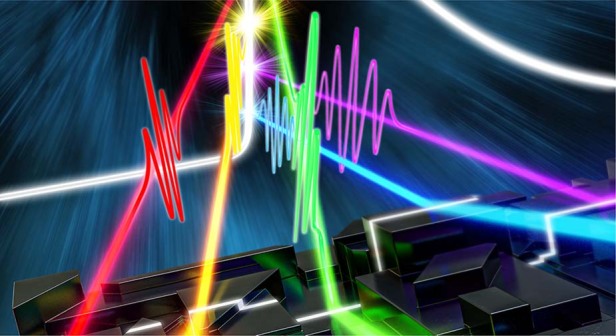

Optical imaging and metrology are crucial in modern science and technology, with applications ranging from on-chip vibration modes measurements^1^ through in vivo biomedical imaging^2,3^ to LiDARs for autonomous vehicles^4^. In particular, rapid and accurate imaging of surface profiles in micro- and nano-scale devices is becoming more important for the investigation of both static^5,6^ and dynamic^1,7,8^ properties of such devices.

On the static property side, higher-dynamic-range and higher-throughput dimensional metrology over larger wafer areas is becoming increasingly important in the semiconductor industry with the advances in microelectromechanical-system (MEMS) devices and three-dimensional stacked integrated circuits (3D-ICs) based on vertical interconnects with micro-bumps and through-silicon-vias (TSVs)^9^. So far, surface metrology using interferometry^10–14^ and confocal microscopy^15,16^ have been utilized, however, each method has limitations in terms of measurement range (typically less than few micrometres) and speed (typically taking hundreds seconds for imaging 1024 × 1024 pixels FOV), respectively.

On the dynamic property side, accurate characterization of vibrations and dynamic behaviours in micro- and nano-mechanical devices is critical for understanding the underlying physics and advancing their applications. In particular, recent emergence of various nonlinear, transient, and complex mechanical dynamics, such as anharmonic vibrations in micro- and nano-mechanical resonators^17–20^, pulsed optomechanics^21,22^, phononic frequency combs^23^, ultrasound sensors^24^, and optomechanical solitons^25^, to name a few, has necessitated real-time surface deformation imaging with finer axial and lateral resolutions, higher speed, and higher dynamic range. Coherent interferometers and white-light interferometers have been widely utilized due to their nanometric axial resolutions and reliability, however, they suffer from sub-micrometre ambiguity range^11,13^ and slow (few-Hz) video rate^26^, missing motions faster than ~metre-per-second. While laser Doppler vibrometers (LDVs) with a bandwidth over GHz can be used to measure high-speed vibrations, they only offer out-of-plane vibration properties at a single spatial point^27^. As a result, vibrations and mechanical motions in micro-scale devices have mostly been characterized by the combination of multiple methods^17,28–31^ or in a stroboscopic way^1,27,32,33^, and real-time multi-dimensional observation of nonlinear or transient dynamics has remained particularly challenging. Direct time-resolved surface imaging of fast mechanical motions has therefore been a missing element in the investigation of full-field dynamic behaviours in micro- and nano-scale devices and mechanical resonators.

Here, we have established a new class of line-scan time-of-flight (TOF) camera technology that can capture both static and dynamic properties of micro-scale devices with high dynamic range. Our method can simultaneously detect TOF changes of >1000 spatial coordinates over several millimetres field-of-view (FOV) with a unique combination of pixel-rates (up to 260 megapixels/s), axial resolution (down to 330 pm) and dynamic range (i.e., 20 log(measurable range/achievable precision)) up to 126 dB. This unprecedented combination of performances enables not only fast and precise imaging of complex structures without requiring much prior knowledge but also real-time observation of fast and non-repetitive mechanical motions in micro-scale devices and mechanical resonators.

## Results

### Operation principle of the line-scan TOF camera

**Fig. 1:**
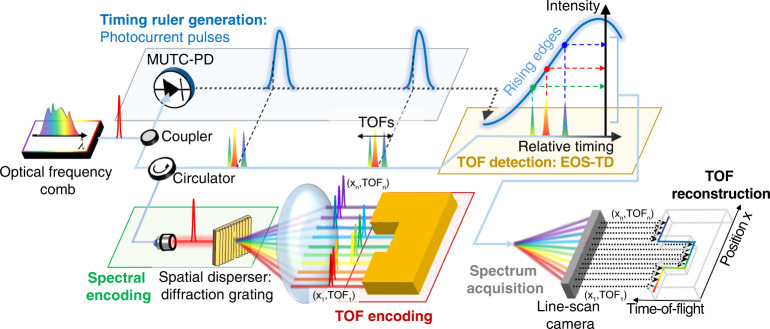
Operation principle of the electro-optic sampling-based line-scan TOF camera. A mode-locked Er-fibre oscillator is used as the optical frequency comb source. For the timing ruler generation, an MUTC-photodiode is used to generate ultralow-jitter photocurrent pulses. For the target imaging, the optical pulses are expanded and spectrally dispersed for space-to-wavelength encoding. After reflected from the target, the TOF-encoded sub-pulses are collected and applied to an EOS-TD for TOF-to-intensity conversion. The EOS-TD output spectrum is analysed with a line-scan camera to simultaneously reconstruct TOF profiles of >1000 spatial points

**Fig. 2:**
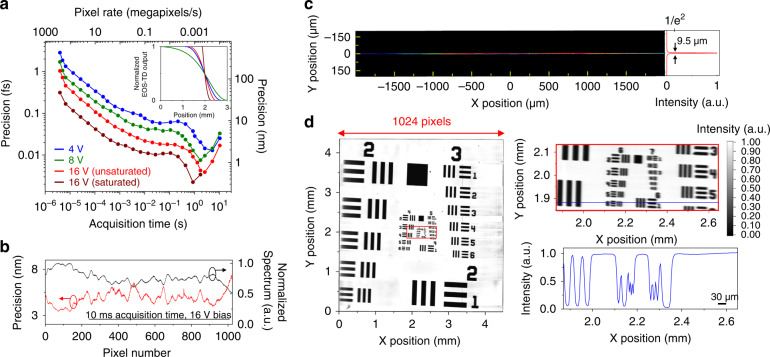
Axial and lateral resolution analysis of the line-scan TOF method. **a** TOF precision measurement in terms of overlapping Allan deviation as a function of acquisition time. Three MUTC-photodiode bias voltages of 4, 8 and 16 V (unsaturated and saturated camera conditions) are demonstrated. Inset: The normalized EOS-TD outputs with respect to the relative timing between the optical pulses and the rising edges of photocurrent pulses show that the measurable ranges of 4, 8 and 16 V (unsaturated) and 16 V (saturated) bias voltages are 3 mm, 1.6 mm, 1.2 mm and 0.4 mm, respectively. **b** TOF precision measurement at each pixel position at 10 ms acquisition time (16 V bias, unsaturated camera). **c** Measured beam profile when focused with 30-mm focal length (see Methods). **d** Microscopic imaging of the resolution target. The top right panel shows the magnified image of groups 6 and 7 (red box of the left panel). As shown in the single line-scan trace, the three bars of Element 6 in Group 6 are decomposed with ~23% contrast, resulting in ~114 lp/mm (4.38 μm) lateral resolution

The operation principle of the demonstrated line-scan TOF camera is shown in Fig. 1. Space-to-wavelength encoding^11,34,35^ with a broad spectrum of a mode-locked Er-fibre optical frequency comb is used to accomplish the line-scan illumination with few-micrometre lateral resolution along several millimetres range. The spectrum of an optical frequency comb (1537–1577 nm in this work) is spatially dispersed by a diffraction grating to encode one-dimensional spatial coordinates into the wavelengths. The beam size incident on the diffraction grating is expanded until its spectral resolution reaches ~0.038 nm, resulting in ~1000 resolvable sub-pulses from ~40 nm bandwidth of the frequency comb, which corresponds to the pixel number of the used line-scan camera (1024 pixels in this work, see Fig. S8). When focused on a sample, the dispersed sub-pulses generate a long and narrow line with 9.5-μm width and >4.4-mm length (Fig. 2c), wherein each sub-pulse undergoes different TOF corresponding to the instantaneous surface profile. Note that, while the space-to-wavelength encoding was recently used for other frequency comb-based imaging methods, they suffered from low pixel-number and axial resolution of tens of micrometres^34–37^ due to the degraded temporal confinement of sub-pulses. To overcome this limitation, we employed an electro-optic-sampling-based timing-detector (EOS-TD)^38–41^ for parallel detection of the TOFs of sub-pulses (see Supplementary Note 1, Fig. S1 and refs. ^37–40^ for more information on the operation principle of the EOS-TD). Here the EOS-TD can detect the relative timing and TOF changes between optical pulses and frequency-locked periodic electric waveforms, such as photocurrent pulses derived from high-speed photodetection^42^ or timing-synchronized microwave signals^38,40,43^, with attosecond-level resolution. While the EOS-TD was recently used to measure the single-point TOF of ~300-fs-long optical pulses with sub-nanometre resolution^44^, in this work, we showed that sub-nanometre axial resolution can be obtained for sub-pulses with >90 ps pulse width as well, allowing massively parallel TOF detection of >1000 sub-pulses over a several mm-long horizontal coordinate.

When the reflected sub-pulses are applied to the EOS-TD, a unidirectional electro-optic phase modulator inside the differential-biased fibre Sagnac-loop phase-modulates them with respect to the timing ruler provided by the periodic electric waveform, resulting in the conversion of their TOF variations into the spectral intensity variations at the Sagnac-loop output. In this work, the rising edge of photocurrent pulses extracted from a modified-uni-travelling carrier (MUTC) photodiode or the microwave signal generated from a frequency-locked voltage-controlled oscillator (VCO) were employed as the periodic electric waveform for realizing a precise yet long-range timing ruler of optical pulses. The EOS-TD converts the timing error between each sub-pulse and the centre of the timing ruler signal into a parallel intensity change at the EOS-TD output via the Sagnac-loop action. As the 1024-pixel InGaAs line-scan camera analyses the optical spectrum of the EOS-TD output, the TOF profile of more than 1000 resolvable positions is simultaneously reconstructed by mapping the spectral wavelength and intensity into the position and TOF, respectively. Note that, although the divided sub-pulses have >90 ps pulse width due to their ~0.038 nm wavelength resolution, the electro-optic sampling process in the EOS-TD still enables sub-nm precision while maintaining the few-micrometric spatial resolving power.

### Axial and lateral resolutions

Figure 2a shows the measured axial precision performance in terms of overlapping Allan deviation. The axial precision is tested under different bias voltages applied to the 22-GHz MUTC photodiode. Higher bias voltage causes photocurrent pulses to have a sharper rising edge, resulting in 3, 1.6, and 1.2 mm measurable range for 4, 8, and 16 V bias voltages, respectively (see inset of Fig. 2a). Due to the sharper rising edge, the axial precision can be improved with higher bias voltage at the expense of slightly narrower measurable range. At 3.9-μs acquisition time (260 megapixels/s pixel rate), which is the shortest acquisition time of the used line-scan camera, the axial precision under 16-V bias voltage is 155 nm. Note that, as the EOS-TD can output the spectrum-encoded TOF profile with every pulse repetition period (4-ns in this work), the maximum acquisition rate can be improved with a faster line-scan camera. As increasing the acquisition time (i.e., data accumulation or increasing exposure), the averaging effect enables precision improvement until slow timing drift caused by thermal drift between mismatched optical fibre length, power drift of the used comb source and timing drift between optical pulses and electric timing-ruler signal kicks in; when using a 16-V bias voltage, the best precision of 580 pm can be obtained at 2.6-s averaging time (394 pixels/s). When such high-precision performances are combined with >mm measurable ranges, large dynamic-ranges up to 126 dB (=20 log(1.2 mm/580 pm)) can be realized. The achievable axial precision at each pixel is limited by the signal-to-noise ratio (SNR) of the camera background noise (white noise). As a result, as shown in Fig. 2b, the achievable axial precision at each pixel has a spectral intensity dependence, ranging from 3.5 nm to 7.4 nm for 10-ms acquisition time (~100 kilopixels/s pixel-rate) under 16-V bias condition. Note that, when the optical spectrum is non-uniform, the precision and dynamic range performances become non-uniform as shown in Fig. S13.

Note that, by saturating the camera, the precision performance can be further improved while slightly sacrificing the measurable range. For example, as shown in Fig. 2a, when increasing the camera electrical gain by 5 times, the camera becomes saturated when the relative timing exceeds ~400 μm (when 16 V bias is used). Due to the increase in TOF detection sensitivity, 47 nm precision can be obtained at 3.9 μs acquisition time (260 megapixels/s), which is a ~3 times improvement. By accumulating data points, the best precision reaches 330 pm at 0.87 s acquisition time (~1.2 kilopixels/s). Since the SNR is almost maintained, the dynamic range is maintained at ~122 dB (=20 log(400 μm/330 pm)).

When a lens with 30-mm focal length (f) is used, the beam sizes (1/e^2^) of the sub-pulses are measured to be ~9.5 μm, and the total horizontal field-of-view (FOV) reaches >4.4 mm (Fig. 2c). A resolution test target (1951 USAF) is used to assess lateral (spatial) resolution. The measured 2D image of the normalized return power spectra is shown in Fig. 2d when the target is laterally scanned along the Y direction. As the patterns of Element 6 in Group 6 are resolved with ~23% contrast, the spatial resolution is determined to be ~114 lp/mm (4.38 μm)^45^. Note that the lateral resolution and horizontal FOV can scale with the effective focal length.

### Surface profile imaging and step heights measurement results

**Fig. 3:**
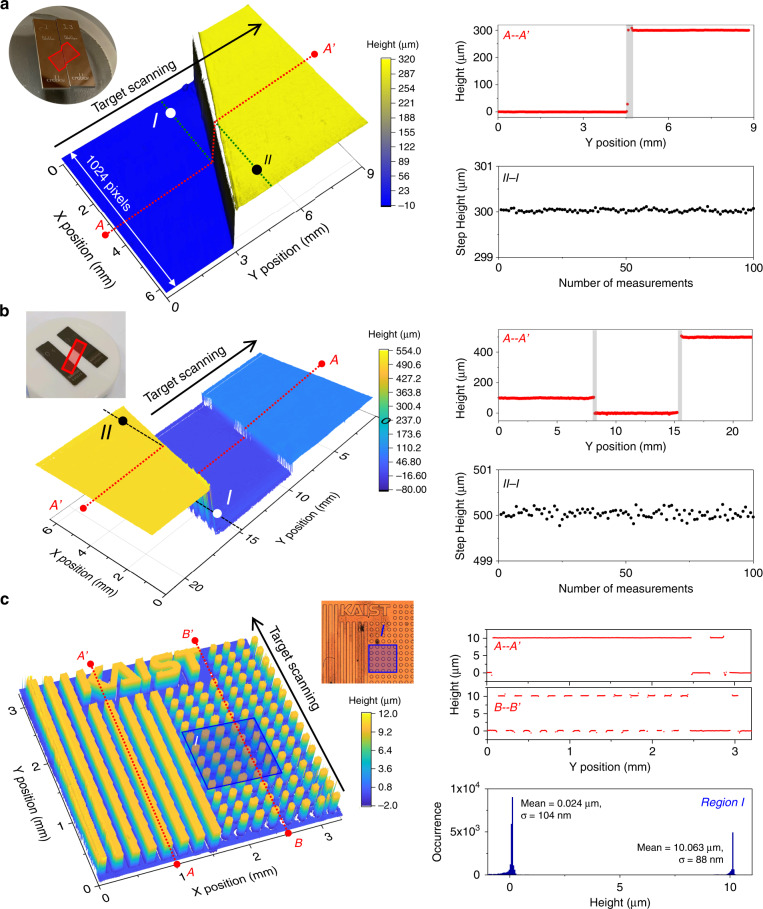
3D surface profile imaging results. **a** Surface profile imaging of two gauge-blocks of the same material (chromium carbide). As shown in the cross-sectional view from point *A* to *A’*, the 300-μm step height is clearly measured. The grey regions indicate the edge of the gauge blocks, where TOF has ambiguity due to reflections from two surfaces. The step height (between points *I* and *II*) is determined to be 300.029 μm with repeatability (the standard deviation value of 100 consecutive measurements at 100-μs acquisition time) of 31 nm and error of +31 nm from a calibrated interferometer result. **b** Imaging results of different materials assembly; two steel gauge blocks attached on a ceramic optical flat. The measured 500-μm step height (*II - I*) show 93-nm repeatability (100-μs acquisition time) and −22 nm error from a calibrated interferometer result. **c** Surface profile imaging of a complicated periodic structure (silicon sample coated with 100-nm-thick silver). A pair of *f* = 60 mm lenses are used for better spatial resolution. A histogram of TOF points in region *I* shows 10.039 μm mean height difference, which has −14 nm error from a confocal microscopy result. Inset: microscopic image (2.5X) of the sample

By rapidly scanning the target in the direction perpendicular to the line (Y direction) using a motorized stage, both ultrafast 3D imaging of surface profiles and precise measurement of step heights can be achieved using the line-scan TOF camera. First, a gauge block assembly with a 300-μm step height made of the same material (chromium carbide) is imaged as shown in Fig. 3a. At 10-kHz line-scan rate (10 megapixels/s pixel-rate), the step height is determined to be 300.029 μm with repeatability (1–σ) of 31 nm for 100 consecutive measurements. To assess the accuracy of the measurement, a calibrated interferometer^46^ with an expanded uncertainty of 40 nm (*k* = 2, level of confidence 95%) is used, and the determined central length is 299.998 μm. The +31 nm error is within the uncertainty due to the flatness of gauge blocks. The demonstrated TOF camera can also measure the surface profiles and step heights of structures made of different materials with different reflectance. Two steel gauge blocks are wrung on a ceramic optical flat, as shown in Fig. 3b, with a reflectance difference of >5 times. The two step heights of 100 μm and 500 μm are clearly measured, with 86 nm (93 nm) repeatability at 10-kHz acquisition rate (10 megapixels/s pixel-rate) for 100 consecutive measurements of the 100-μm (500-μm) heights. The determined step height shows +15 nm and −22 nm errors from the calibrated interferometry results (100.014 μm and 500.060 μm). Note that, compared to Fig. 3a, the repeatability is slightly degraded due to the lower reflected optical power from the ceramic optical flat, which resulted in a degradation in TOF measurement precision. High axial and lateral resolutions also allow for precise profiling of more complicated structures. As shown in Fig. 3c, a silicon sample coated with 100-nm thick silver is prepared as a periodic structure with trenches and pillars of 100-μm width and ~10-μm height, repeated with 100-μm spacings. The mean step height (difference between two mean heights) is measured to be 10.039 μm, which is −14 nm off from a confocal microscopy result (10.053 μm, see Fig. S2).

Note that, for accuracy and repeatability evaluations, the 3D imaging results shown in Fig. 3 were obtained by synchronizing the line acquisition and motor movement: each line data is acquired once the motor has settled down to avoid motor vibration-induced uncertainty. Higher-speed 3D imaging can be achieved by rapidly scanning the target without synchronization. When the gauge-blocks are rapidly scanned (with the motor’s fastest scanning speed; 0.7 m/s), as shown in Fig. S3, the 3D imaging takes just 3.47 ms to analyse a 6.4 mm × 2.4 mm region with 1024 × 882 pixels resolution. Note that the achieved pixel rate (260 megapixels/s) corresponds to >120 Hz frame rate (3D image refresh rate) 3D profile recording in full high definition (FHD, 1920 × 1080 pixels resolution). Although the target is scanned stepwise in this study due to the mechanical limitations of the motor, the acousto-optic deflection^13,47^ may be employed to enable vibration-free and high-speed scanning in the future.

### Real-time dynamic imaging results

**Fig. 4:**
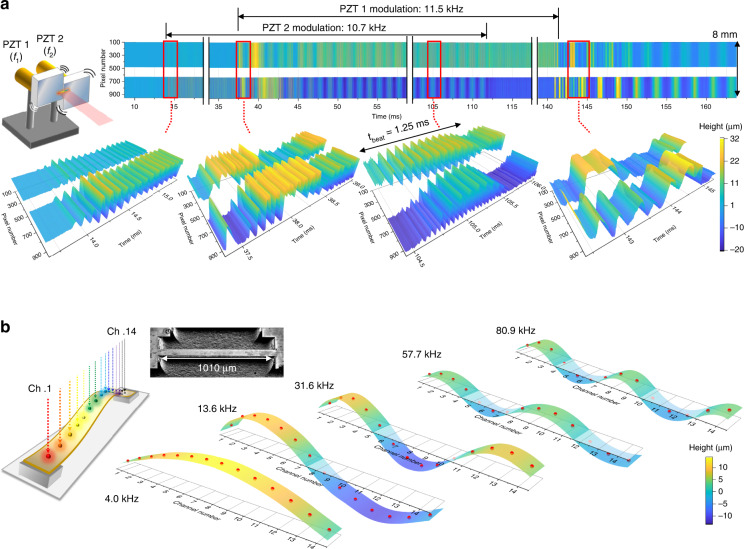
Dynamic imaging results. **a** Interaction between two PZT-attached mirrors. *f* = 75 mm lens is used for ~10 mm horizontal FOV. The two PZTs are driven for ~100 ms durations with ~25 ms delay. The lower panels illustrate reconstructed TOF traces at the moment of modulation start, interaction transient, steady-state, and end of modulation in sequence. The entire TOF trace record can be found in Supplementary Video 1. **b** Real-time observation on MEMS bridge’s flexural mode shapes. 14 beams with ~8 μm beam size and ~880 μm FOV are incident along the length of the bridge (see Methods). The resonant motions of first five flexural modes (from 4.0 to 80.9 kHz) are measured. TOFs of 14 local positions are indicated by red dots and TOF profile between dots is interpolated with the spline method. Inset photo shows the scanning electron microscope image. The entire TOF trace record can be found in Supplementary Video 2

The inertia-free scanning and large dynamic-range capabilities enable us to capture ultrafast mechanical motions with >400 m/s speed. As the first example of functionality validation, the interaction between two piezoelectric transducer (lead zirconate titanate, PZT)-mounted mirrors that are firmly fastened on a rigid plate (Fig. 4a) is observed. When the two mirrors are driven at resonances of 11.5 and 10.7 kHz, respectively, their interaction builds a beating pattern repeating every 1.25 ms at steady-state. As shown in Supplementary Video 1, the two mirrors’ interacting motion is composed of irregular and multi-harmonic motions, yet can be readily analysed without requiring data accumulation or entire movement repetition^1,22,33^. The complex mode coupling between two PZT-mounted mirrors’ multi-harmonic modes could also be observed in real-time (see Fig. S14 for more detailed information and analysis). During the interaction, the instantaneous speed reaches up to ~4 m/s, which can make accurate distance determination difficult in interferometry-based methods. Although there exists just a few ms of non-repetitive transient before settling down to the steady-state, the demonstrated line-scan TOF camera allowed real-time observation of the transient motions (Fig. 4a).

As another example of real-time dynamics capture, we performed the dynamic imaging of mechanical motions of MEMS bridge (i.e., double-clamped beam) device. By aligning the line beam in bridge’s longitudinal direction, various resonant motions can be observed in real-time. For this experiment, a 1010-μm long and 90-μm wide silicon micro-bridge is prepared (see Methods). In order to reduce rotating motion-induced (or round surface-induced) path length error (Fig. S16) and enhance TOF detection accuracy, instead of a diffraction grating, we designed and implemented another space-to-wavelength encoding device by combining a dense wavelength division multiplexing (DWDM) coupler and fibre bundle (see Methods and Fig. S4). When spectrally divided beams are focused on the bridge under test, the 14 beams (14 DWDM channels) are irradiated to the sample with ~6.7 μm beam size and ~70 μm spacing (Fig. S4). As shown in Fig. 4b and Supplementary Video 2, five flexural modes of the bridge (4.0, 13.6, 31.6, 57.7, and 80.9 kHz) could be clearly observed. At resonances, the bridge oscillates with amplitudes ranging from hundreds nm to tens μm, and the instantaneous speed of some spatial points exceeds 5 m/s, the motions of which are difficult to measure when using conventional methods (both interferometry- and microwave-based methods). Note that all resonant motions can be observed in real-time with high-resolution by recording TOFs of 14 spatial points on the bridge, and stroboscopic detection (i.e., synchronizing data acquisition with bridge motion^22^ or beam scanning^32^) was not required.

## Discussion

By combining space-to-wavelength encoding, electro-optic sampling-based timing detection, and high-speed line-scan camera, we have demonstrated a new type of TOF camera that can achieve ultrahigh-pixel-rate, massively parallel sub-nm-precision TOF detection with few-μm lateral resolution over several mm FOV. Performance comparisons (in terms of axial resolution versus detectable range and axial resolution versus pixel-rate) with other state-of-the-art parallel 3D imaging techniques are presented in Fig. S5.

The demonstrated TOF camera can image static surface profiles with high precision and accuracy, as well as capture fast and large-range surface deformations in micro-scale mechanical resonators in real-time. As a result, we believe that the demonstrated TOF camera will become a vital imaging tool for studying various vibrations and dynamic behaviours in micromechanical devices, including nonlinear, transient, and complex mechanical dynamics^17–20^, which have been difficult to measure with previous approaches. Some examples include capturing of non-repetitive transients and observation of various mode-shapes.

Since the current acquisition rate and precision are limited by the speed of spectrum-analysing camera, the performances might be further improved by using a fast photodiode array with higher linearity^48,49^. Further, our TOF camera may be also expanded to full 3D dynamic imaging in the near future by employing a 2D-array of fibre bundle^50^, a combination of virtually imaged phased array (VIPA) and grating^11,51,52^ or a MEMS-mirror system^30^ for generating a spectrum-encoded 2D illumination on a sample.

## Methods

### System configuration of line-scan TOF camera

A 250-MHz mode-locked erbium-doped fibre oscillator was used as an optical frequency comb source. The comb signal was amplified by an erbium-doped fibre amplifier (EDFA) to ~50 mW, and split into two paths. One path is used for generating a timing ruler signal. Optical pulses with ~5 mW average power is applied to a 22-GHz MUTC-photodiode to generate a timing ruler signal (Fig. 1). Note that other methods can be used to generate the timing ruler signal, for example, a microwave voltage-controlled oscillator (VCO) synchronized to the comb source^38–40^ (Fig. S6) or a microwave signal by band-pass filtering the photocurrent pulses (Fig. S7). Note that, while most measurements in Figs. 3 and 4 were conducted with rising edges from the MUTC-photodiode, the phase-locked VCO generated sinusoidal timing ruler signal for the measurement in Fig. 4b. The other path is used for applying the line beam to the target after spectrally dispersed for space-to-wavelength encoding. When ~40 mW of optical power is incident on the reflective surface (e.g., a gauge block), ~8 mW of reflected power is collected. The collected sub-pulses containing TOF information are split by a coupler and applied to an EOS-TD (~80%) and a power monitor (~20%) for TOF detection and sensitivity calibration, respectively (see Supplementary Note 2). Finally, the EOS-TD output spectrum is measured by an InGaAs line-scan camera (Manx SQ 1024, Xenics) to reconstruct the TOF profile along the FOV. A ~1 m-long dispersion-compensating fibre with a polarization controller was inserted before the EDFA to compensate for group delay dispersion (GDD). The remaining GDD after compensation was −0.037 ps^2^, resulting in ~1.1 ps timing delay across ~40 nm spectrally spanned sub-pulses. Note that, in our system, the EOS-TD concurrently detects the entire timing of the sub-pulses, each of which is labelled by different wavelength, in a parallel way; as a result, the temporal overlapping between sub-pulses has no impact on system performance (such as precision, accuracy and measurement speed) and our method is free from crosstalk issue. By minimizing the GDD, all the sub-pulses are located at near-centre position of the rising edge. The remained relative timings between sub-pulses (<1.1 ps) can be compensated by using a look-up table method. The actual EOS-TD and camera responses over entire sub-pulses (that include the information on residual timing offset between different sub-pulses) can be simultaneously recorded when a flat mirror is scanned. This allows for the construction of a look-up table that will subsequently be utilized for the calibration of the actual TOF measurement result. The entire system is also mounted on a rubber plate and made airtight by a polycarbonate case for isolation from ambient vibration and sound. The isolation from ambient noise sources can reduce the amount of slow timing drift, enabling the group delay of each sub-pulse not to change during measurement.

### Space-to-wavelength encoding

When a fibre-coupled collimator collimates a ~2.15 mm diameter beam, two pairs of dual doublet lenses expand it ~6 times to be ~12.9 mm. Here, to minimize aberration, two identical doublet lenses with sufficiently large aperture are combined closely; two lenses of *f* = 35 mm and 1” diameter, and two lenses of *f* = 250 mm and 2” diameter are used. The expanded beam is incident on a transmission diffraction grating (1200 grooves/mm) at an angle of 68 degrees. As the large angle-of-incidence makes the beam size to be ~34.4 mm along the groove direction, the spectral resolution of the grating becomes ~0.038 nm at 1557-nm centre wavelength. Finally, the spectrally dispersed beam is focused on a target surface (or photodiode array in line-scan cameras) using a doublet lens (or a pair of doublet lenses), mapping the spatial coordinate into the wavelength. For example, when a *f* = 50 mm lens is used, the calculated pixel separation is ~6.15 μm, and the beam size (1/e^2^) at focus is measured to be 14.1 μm. The horizontal FOV for a 38-nm bandwidth signal is ~6.37 mm, resulting in ~1036 resolvable spatial (spectral) points. The RMS wavefront error is calculated to be <0.05 waves.

### Evaluation of TOF detection precision

To evaluate the TOF detection precision (Fig. 2a), the relative timing fluctuation between optical pulses and rising edges is monitored in the absence of the sensor head part. The optical pulses are located at the middle point of rising edges, where the TOF detection sensitivity is maximized. The relative timings of 1024 sub-pulses (i.e., pixel number of line-scan camera) are measured at a 254-kHz line rate, which is the maximum acquisition speed of the line-scan camera. After consecutive TOF measurements, the precision is determined by calculating overlapping Allan deviations against different acquisition times.

### 3D surface profile imaging methods

To make the gauge block assembly (Fig. 3a), two chromium carbide gauge blocks with nominal heights of 1 mm and 1.3 mm were wrung on an optical flat to make a step height of 300 μm. The sample in Fig. 3b was fabricated by wringing two steel gauge blocks with nominal heights of 100 and 500 μm on a ceramic flat. The sample shown in Fig. 3c was fabricated by etching a silicon wafer, and a 100-nm thick silver film was coated for high reflectance. Depending on the target structure, different focusing lenses were employed. For example, when a large FOV is required as in Fig. 3a, b, a *f* = 75 mm lens is used for ~10 mm horizontal FOV. When a small beam size and high lateral resolution are required for imaging complex structures (in Fig. 3c), a pair of *f* = 60 mm lenses is used to reduce the beam size to below 10 μm. A pair of *f* = 60 mm doublet lenses are used instead of a *f* = 30 mm lens to minimize the aberration. The target is scanned along the Y direction (perpendicular to the dispersion direction) by a precision motor stage (±100 nm repeatability).

For gauge block assembly imaging in Fig. 3a, b, the acquisition time of line-scan camera was set to be 5 μs and 50 spectra were averaged to determine one line-TOF (250 μs/line rate). For periodic structure imaging in Fig. 3c, the acquisition time was increased to 14 μs due to the low optical reflectance at the sample, and 20 spectra were averaged (280 μs/line rate). To obtain the 3D imaging results shown in Fig. 3, the motor movement was synchronized with the line-scan camera acquisition to assess complete 3D imaging performances. Note that, for each line, in order to settle down the motor vibrations, a ~18 ms break was inserted between every motor movement. Because the 3D image in Fig. 3a consists of 294 lines, the total imaging time for gauge block assembly was ~5.4 s (294 lines × 18.25 ms/line) when the TOF measurement itself took only 0.074 s; the total measurement time was mostly limited by the break times in motor movements. As shown in Fig. S3, fast 3D imaging can be realized by acquiring line spectrum while motor continuously scanning the sample with 0.7 m/s speed without synchronization. In this case, the 3D imaging took only 3.47 ms to analyse a 6.4 mm × 2.4 mm region with 1024 × 882 pixels resolution when the line camera measures line-TOF profiles with 254 kHz line rate.

The TOF measurement of round-shape surfaces or rotational motions is challenging for two reasons: first, the optical power does not return after reflected from the sample due to the limited numerical aperture (NA) and second, the grating induces additional optical path when the reflected surface has a curvature or rotates. Figure S15 shows the measurement results for a round-shape sample (e.g., micro-solder bumps), illustrating the impact of limited NA. There is little power return at the boundaries of bumps and only several pixels at the top-centre could acquire power return. As a result, the TOF could be measured for 3–6 pixels at the centre of the bumps. The measured TOF at the centre position of three bumps have a fairly good agreement. Note that the measurable region can be expanded by increasing the NA of lens. Even in the case when the optical power can be returned from the round surface (for example, by using larger NA), the returned optical path length can be changed by the grating. Figure S16 shows such situations where the returned path length varies at the grating. Due to the asymmetry of grating, the optical path length is changed and returned optical power is decreased when the target rotates or has a slope. In order to avoid such path length change problems, when measuring the flexural mode shapes of a bridge shown in Fig. 4b, instead of a grating, we used a DWDM-based fibre bundle for spectral encoding.

### Two PZT-mounted mirrors imaging method

For imaging the interaction between two PZT-mounted mirrors (Fig. 4a), two identical PZT stacks (P-840.20, Physik Instumente) are attached to optical posts of different lengths and then fastened to an aluminium plate. Two square silver mirrors (1” in length) are attached to the PZT stacks asymmetrically to resonate at different frequencies. The spacing between the two mirrors (which is the unmeasured region between pixels 500 and 630 in Fig. 4a) is ~1.3 mm. A two-channel arbitrary waveform generator (AWG) drives two PZTs with ~100 ms-long sine waves of 11.5 and 10.7 kHz. The PZT2 is driven first by the 10.7 kHz signal, and then the PZT1 is driven at 11.5 kHz with a ~25 ms time delay. The ~10-mm-long beam illuminates the two mirrors.

### Silicon micro-bridge sample preparation

Silicon micro-bridges are fabricated for mode shape observations (in Fig. 4b). The microfabrication process of the silicon bridge starts with a 6-inch silicon-on-insulator (SOI) wafer of a 2 ± 1 μm silicon device layer, a 1 μm ± 5% buried oxide (BOX) layer, and a 500 μm-thick (100) silicon handle. First, a positive photoresist is spin coated on the front side and exposed to UV for bridge patterning. Silicon is then selectively removed by deep reactive ion etching (DRIE). The SiO_2_ layer exposed after Si bulk etching is also removed by RIE, followed by removal of the photoresist. Once the micro-bridge patterns are defined, xenon difluoride (XeF_2_) isotropic etching of the Si handle is performed to make the bridges suspended. Note that the photoresist is spin coated and patterned again to cover the top surface and side walls of the patterned silicon device layer for protection during XeF_2_ etching. After XeF_2_ etching, the protection photoresist is stripped and the SiO_2_ layer underneath the Si layer is removed by wet etching using buffered oxide etchant (BOE), leaving arrays of Si micro-bridges. As the final step, a 30 nm Al-1at.%Si alloy film is sputter deposited on the silicon bridge to achieve high reflectivity.

### 16-channel fibre bundle for imaging flexural mode shapes

For more accurate (flexural) mode shape observation, instead of a grating, a 16-channel DWDM and fibre bundle are utilized to map optical wavelength into the spatial coordinate. As shown in Fig. S4, a PM-DWDM divides and filters the frequency comb’s broad spectrum into 16 channels and transfers to a 16-channel PM fibre bundle. At the end of the fibre bundle, the 16 fibre ends are aligned and spaced apart by the fibre cladding (~127 μm). Since the width of the entire fibre ends is ~2030 μm, a pair of aspheric lenses is used to reduce it by a factor of 2 (0.5× magnification) yielding a horizontal FOV of ~1000 μm. After the lens combinations and placement (i.e., spacing between the lenses and the fibre bundle) are optimized, the working distance is measured to be ~8 mm and the 16 channels have ~1 mm FOV. When focused, each beam has a beam size of ~6.7 μm (1/e^2^). The 14 beams (note that the EDFA amplification bandwidth covers up to 14 channels) illuminate the bridge longitudinally, and reflected pulses are applied to the EOS-TD for precise TOF-to-intensity conversion. The centre wavelengths assigned to DWDM channel numbers 18, 20, 22, 24, 26, 28, 30, 32, 34, 36, 38, 40, 42 and 44 are 1563.05, 1561.42, 1559.79, 1558.17, 1556.55, 1554.94, 1553.33, 1551.72, 1550.12, 1548.51, 1546.92, 1545.32, 1543.73 and 1542.14 nm, respectively. The resulting spectra are analysed with line-scan cameras.

## Supplementary information

Supplementary Information

Supplementary Video 1

Supplementary Video 2

## Data Availability

The data that support the findings of this study are available from the corresponding author upon request.

## Code availability

The simulation and computational codes for this study are available from the corresponding author upon request.
