# Supplementary material for: Massively parallel electro-optic sampling of space-encoded optical pulses for ultrafast multi-dimensional imaging
Source: Light Sci Appl. 2023 Feb 15;12:44. doi: 10.1038/s41377-023-01077-7 (PMC9932157; doi:10.1038/s41377-023-01077-7)
Supplement: Supplementary file 1 — Supplementary Information [file 41377_2023_1077_MOESM1_ESM.pdf]

Supplementary Information for

"Massively parallel electro-optic sampling of space-encoded optical pulses for ultrafast multi-dimensional imaging"

Yongjin Na, Hyunsoo Kwak, Changmin Ahn, Seung Eon Lee, Woojin Lee, Chu-Shik Kang, Jungchul Lee, Junho Suh, Hongki Yoo, and Jungwon Kim

Supplementary Note 1. Electro-optic sampling-based timing detector (EOS-TD)

The EOS-TD is a timing detector that precisely detects the relative timing between optical pulses and electric waveform. Several types of EOS-TD with different configurations have been reported in various laser-microwave synchronization systems, but in general, EOS-TDs are based on an all-polarization-maintaining (PM)-fibre Sagnac loop interferometer. In this work, an EOS-TD with a unidirectional electro-optic phase modulator (EOM) and a non-reciprocal π/2-phase bias (see Fig. S1) is employed due to its ultralow background noise characteristics. When optical pulses are applied to the Sagnac loop, the EOM phase-modulates the circulating pulses via the instantaneous voltage (proportional to the instantaneous timing) of the EOM-driving electric waveform. When the counter-propagating pulses are combined, and the combined signal is split again at the fibre coupler of the Sagnac loop, the phase differences between the counter-propagating pulses induce a relative timing-dependent intensity variation at the coupler output.

Supplementary Note 2. Calibration of the EOS-TD

The TOF discrimination sensitivity of the EOS-TD is nonlinear due to the rising edges' reduced slope at the ends and the finite V_{pi} of the EOM. To calibrate the EOS-TD's nonlinearity along TOF,

before TOF measurements, a flat mirror is axially scanned by a precision motorized stage (±100 nm repeatability, M-126, Physik Instrumente) to measure the EOS-TD response over the entire measurable range and make look-up tables (LUTs). Fig. S9 shows the measured EOS-TD output spectra when the optical pulses' relative timing is linearly scanned with respect to the rising edges with different bias voltages until detection ambiguity occurs (the total travel ranges are defined as measurable ranges; 3.0 mm, 1.6 mm, and 1.2 mm for 4 V, 8 V, and 16 V bias voltages, respectively). As shown in the left panels of Fig. S9, the nonuniform response along wavelength (pixel) is due to the spectral intensity-dependent sensitivity variation, and for each pixel, the nonlinear response along axial position is due to the nonlinear shapes of rising edges. Using the LUT method, the EOS-TD response is linearized to 4×10^{-6} level, which is the motor's linearity, over the entire measurable ranges as shown in the middle panels of Fig. S9. Due to the EOS-TD's high repeatability, most of the remained nonlinearity lies within the motor's repeatability (±100-nm). The LUTs can be acquired either by scanning the timing of the probing pulses or the photocurrent pulses.

Since the TOF detection sensitivity is proportional to the input optical power to the EOS-TD, the return power variation due to reflectance variation or out-focusing should be addressed while measuring the TOF. To minimize the systematic uncertainty from power variation, ~20% of the returned power is split for monitoring the return spectrum variation. The EOS-TD's response curves and the spectra of split power are measured as shown in Fig. S10. For each pixel (wavelength), the EOS-TD responses at several spectral powers are obtained. When measuring the target, the actual response at each pixel is estimated by interpolating the measured responses. As shown in Fig. S10**c**, the high linearity between input optical power and sensitivity enables accurate calibration of the input power dependence of the TOF detection sensitivity.

As the axial precision performance has power dependence, at lower optical power, several methods can be employed to recover the signal power as well as the precision performance. For example, increasing the exposure time increases signal power at the expense of slower measurement speed. Increasing the camera's electrical gain maintains the measurement speed at the expense of slightly increased noise power and worse precision (Fig. S11). When exposure time and gain are increased to 200 μs and 100 times, respectively, 14-nm precision can be acquired with only ~1 μW of EOS-TD input optical power (equivalently, each pixel is supplied <200 pW) (Fig. S12). The maximum (i.e., the maximum optical power not to saturate line camera) and the minimum (i.e., the minimum optical power enabling TOF detection) sub-pulse's average powers into EOS-TD also depends on the camera operation conditions (see Table S1). When the camera is operated with high camera gain (gain=100) and long exposure time (500-μs exposure time), our method could detect the TOF when sub-pulse with only 0.82 pW power is applied to the EOS-TD.

Two methods were used to measure the spectra of EOS-TD output and the returned optical power: (a) using an optical switch and (b) using two line-scan cameras. For the optical switch method, a PM optical switch with 300-kHz switching speed (NSSW-130110333, Agiltron) is installed before the line-scan camera to switch between the EOS-TD output and the power monitor port. At each line measurement, the line-scan camera measures the spectra of the EOS-TD output and the power monitor in turn. Since this method does not require wavelength-to-pixel calibration between line-scan cameras, the EOS-TD output and the return power spectra have exact pixel correspondence. The 3D imaging results in Fig. 3 are obtained with the optical switch method. However, as this method requires synchronization between motor movement, optical switch and the line-scan camera, it may suffer from motion-blur and imaging speed limitation for dynamic motion recording (Fig. 4) or high-speed imaging (Fig. S3). Thus, two cameras method with two

identical spectrum-acquisition configurations is utilized, where two cameras simultaneously measure the EOS-TD output and return power. This method enables a real-time measurement; therefore, the rapid motions can be measured without motion-blur. Also, since the step-wise movement of the motor stage is not required, the sample can be continuously scanned with maximum motor speed (Fig. S3). Calibration target with equally spaced lines is measured to calibrate wavelength-to-pixel correspondence between the two cameras. Since the target has 50 μm divisions, the uncertainty during two camera calibration is below 50 μm, which is ~4 pixels. A calibration target with smaller spacing can be used for better synchronization.

Supplementary Note 3. The use of different electric waveforms as timing ruler signal

By using different microwave waveforms, the measurable range and precision can be tuned. Since the photocurrent pulses are composed of numerous harmonic frequencies of the comb repetition rate (up to the photodiode bandwidth), any harmonic frequency signal can be extracted as a timing ruler. For example, as shown in Fig. S7, a 1.5-GHz signal (i.e., 6th harmonic frequency) can be generated by an RF band-pass filter. The MUTC-photodiode is driven with 8 V bias voltage and terminated with 50 Ω. The extracted signal is then amplified and band-pass filtered again for removing high-harmonic components. As a result, a 1.5-GHz signal of ~12 dBm power is generated and applied to the EOS-TD as a 100 mm-long timing ruler signal. The probing pulses undergo TOF variation by the precision motor stage (50 mm travel, ± 100 nm repeatability), and an optical delay line is inserted to locate optical pulses at the zero-crossing point of microwave signal. As a result, the 1.5-GHz microwave signal extends the measurable range up to ~50 mm (i.e., half of wavelength, in round-trip), which is ~40 times longer than the measurable range of rising edges. The reduced TOF detection sensitivity linearly scales the precision performance: 5.3 -μm precision at 260 megapixels s^{-1} of acquisition rate, and when averaged, down to 16-nm

precision at 4.7 kilopixels s^{-1}. Since the optical power is adjusted to fully utilize the camera's non-saturation range, the dynamic range performance is maintained to ~130 dB.

An independent microwave source, such as a voltage-controlled oscillator (VCO), can be utilized to generate timing rulers. As shown in Fig. S6, the VCO signal is frequency synchronized to the multiple of the repetition rate. For the mode shape observation in Fig. 4**b**, another EOS-TD is used for ultralow-noise synchronization of an 8-GHz VCO. Using a continuous sinusoidal timing ruler enables TOF detection of sub-pulses present at arbitrary relative timings.

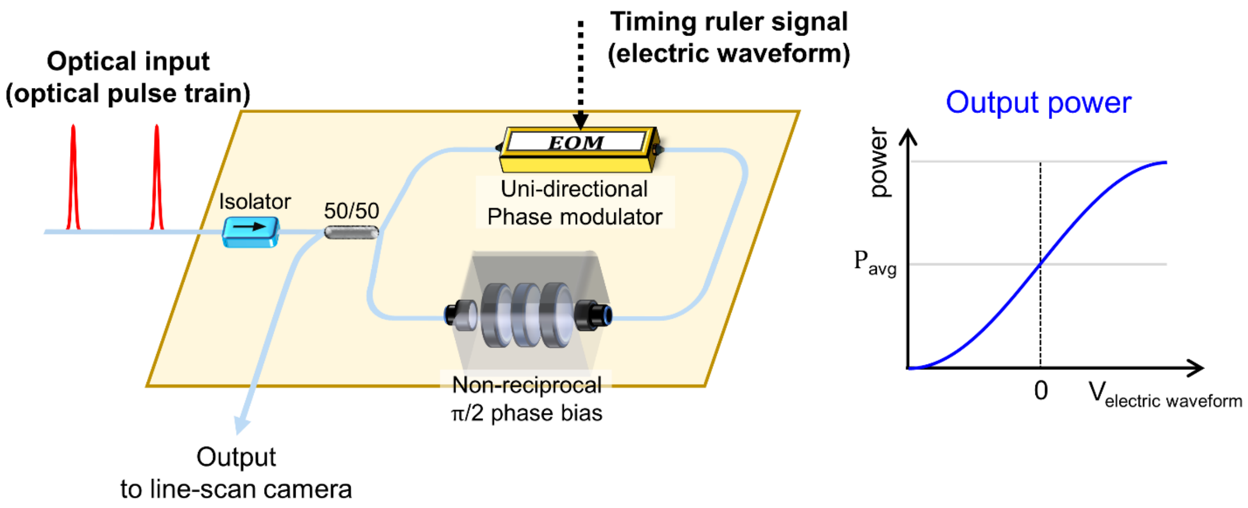

Figure S1 | Structure and operation of the EOS-TD.

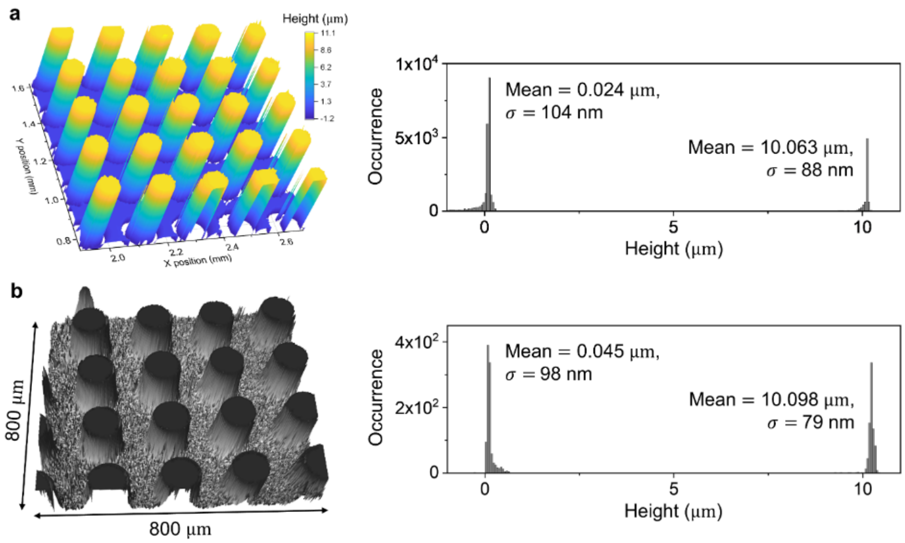

Figure S2 | Comparison of imaging results of region *I* in Fig. 3c with a confocal microscopy. a, Measurement result from our method (a magnified view of region *I* in Fig. 3**c**). The right panel is a histogram of entire data points (50 nm data bin size). **b**, Measurement result from a confocal microscopy. The axial scanning takes ~80 s with 70 nm spacings. The raw 3D image is post-processed to reduce distortions from sample alignment error, lens aberration, and coordinate error.

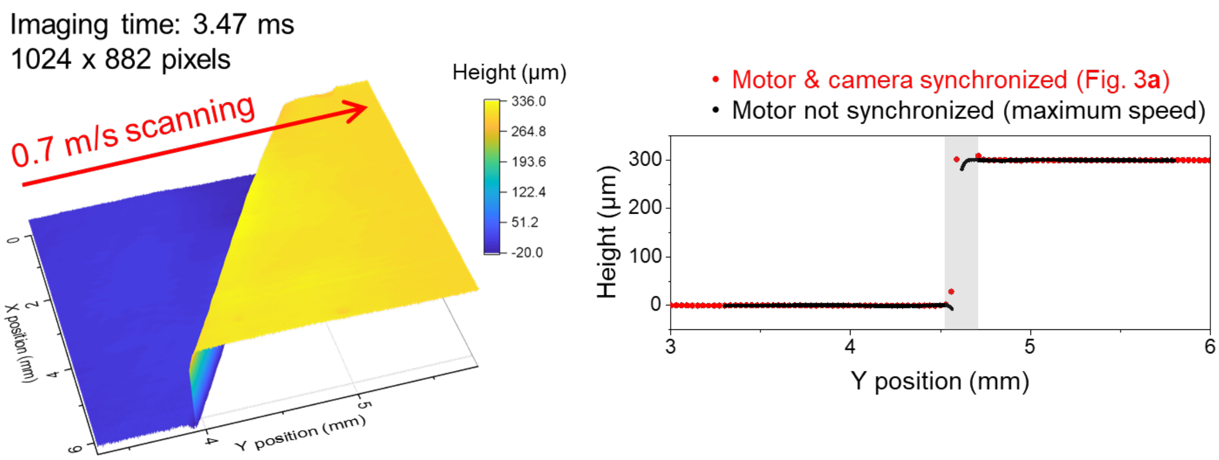

Figure S3 l Rapid imaging result. The gauge block assembly in Fig. 3**a** is rapidly (0.7 m s^{-1}, which is the maximum travel speed of motor stage) scanned without synchronization with line-scan cameras. The line-scan cameras continuously capture the EOS-TD output and return power spectra to reconstruct instantaneous TOF profile with 254 kHz, which is the camera's maximum acquisition rate. The rapid imaging takes only 3.47 ms to analyse the surface profile of 6.4 mm x 1 mm region with 1024 x 882 pixels definition.

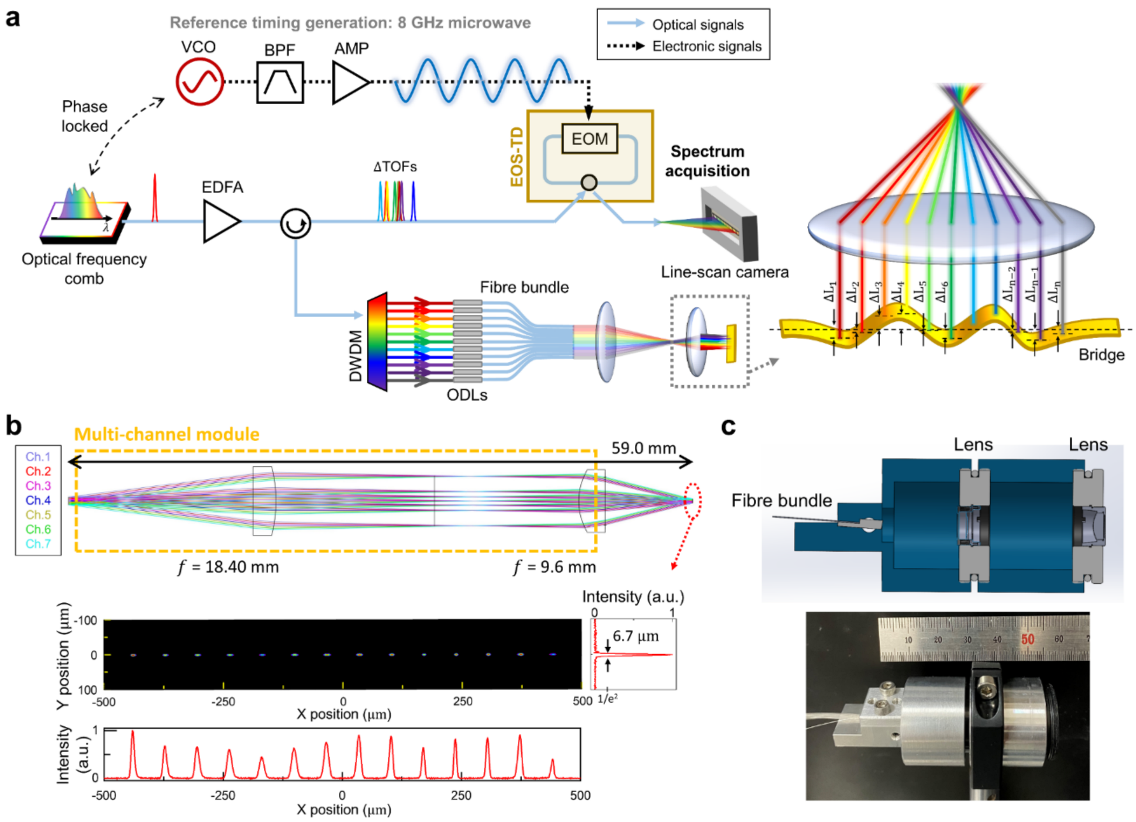

Figure S4 l Schematic diagram of 14-channel parallel TOF detection. a, Experimental setup. An 8-GHz voltage-controlled oscillator (VCO) is used for timing ruler generation. Out of amplified optical spectrum, the DWDM filters 14 channels with 200-GHz channel spacing. At the other end of the fibre bundle, 14 fibre ends are glued with polarization and polished angles aligned, separated by 125 µm. The 14 channels have a total length of 1,750 µm, and a pair of lenses reduces the beams by half, resulting in a horizontal FOV of ~880 µm. **b,** The beam magnification (X0.5) optimization results. The optimization is made with 7 beams. The bottom panel shows the measured beam size of 6.7 µm ($1/e^2$) and the horizontal FOV (880 µm) at the focus. **c,** The structure and photo of the multi-beam illumination module.

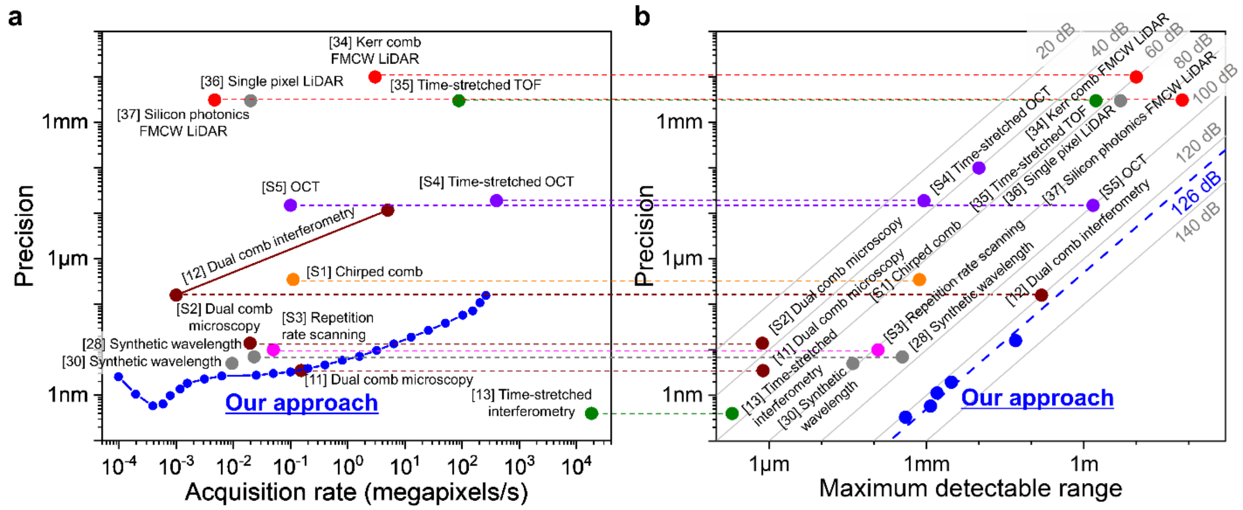

Figure S5 | Performance comparison between the state-of-the-art 3D imaging techniques. a, Precision (overlapping Allan deviation) versus the acquisition time. **b,** Precision versus the maximum detectable range and resulting dynamic range.

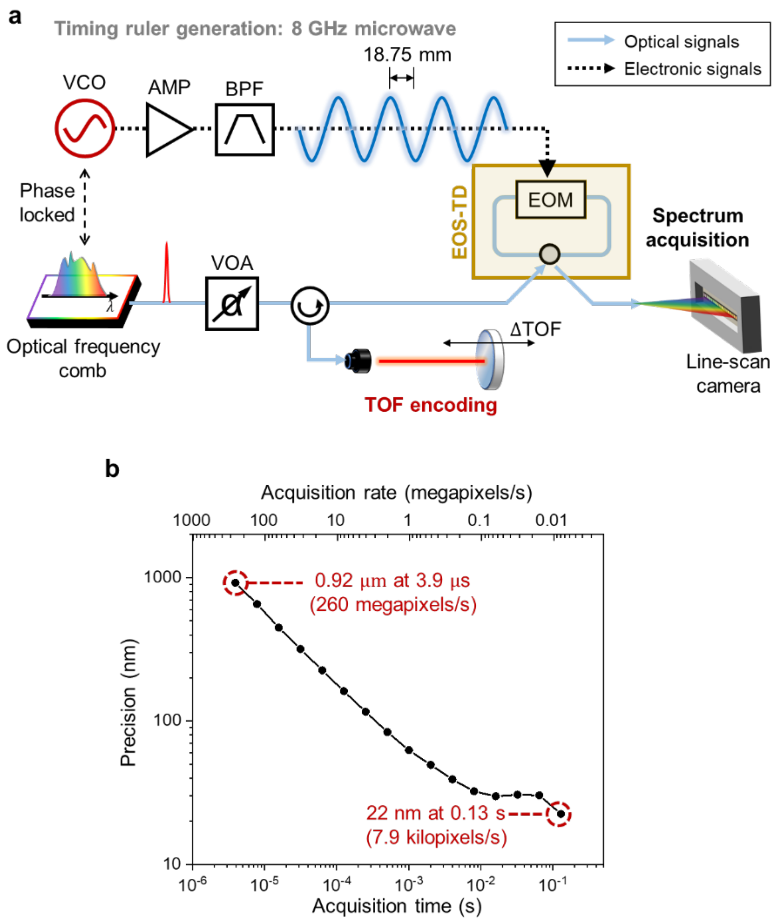

Figure S6 | Schematic diagram when using a VCO signal for a timing ruler. a, Experimental setup. An 8-GHz VCO is used for timing ruler generation (~9.38 mm measurable range). The VCO is tightly synchronized to the comb source using another EOS-TD. VOA, variable optical attenuator; AMP, radio frequency amplifier; BPF, band-pass filter. **b**, TOF detection precision performance.

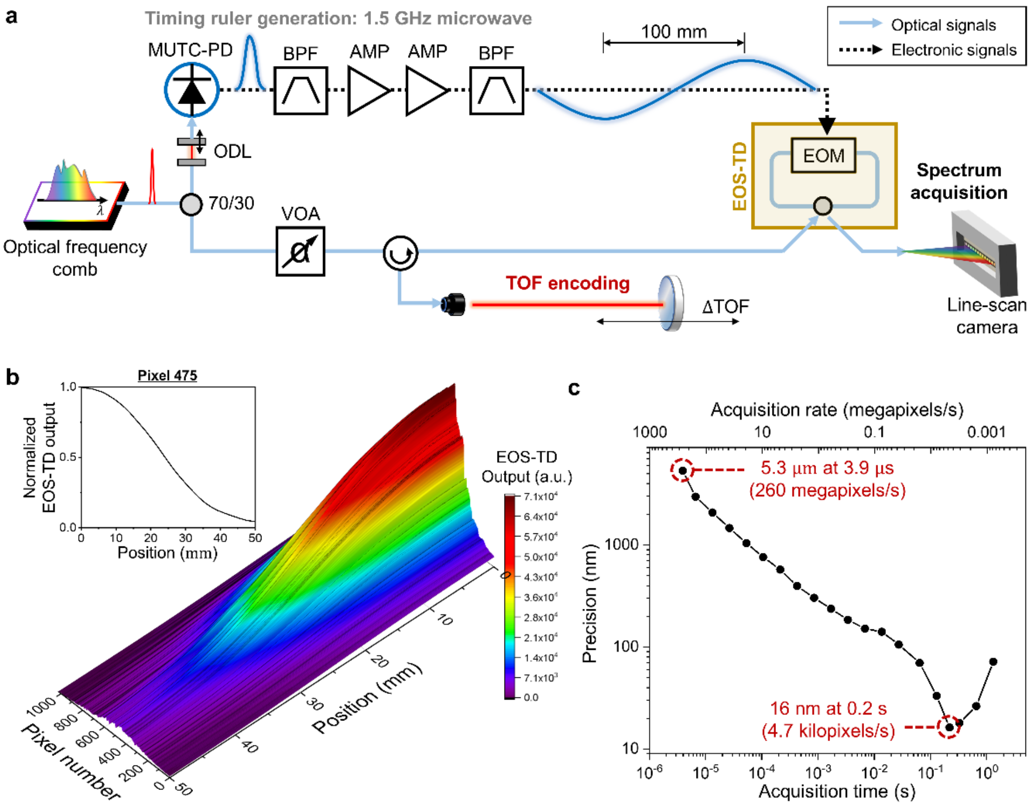

Figure S7 l Measurable range extension by using a bandpass-filtered microwave as a timing ruler signal. a, Experimental setup. The photocurrent pulses from MUTC-photodiode are band-pass filtered (~1.5 GHz centre frequency) and amplified. The 1.5-GHz microwave signal of ~12 dBm power is generated as a ~100 mm-long timing ruler signal. The probing pulses (70% power of optical frequency comb) encode TOF variation induced from a precision motor stage. ODL, optical delay line; BPF, band-pass filter; AMP, radio frequency amplifier; VOA, variable optical attenuator. **b,** EOS-TD output signal as a function of motor position. The return power variation along 50 mm travel is calibrated. The 1.5 GHz microwave signal extends the measurable range (round trip) up to ~50 mm. Inset: Normalized EOS-TD output of the 475th pixel as a function of motor position. **c,** Precision performance. The extended measurable range and reduced TOF detection sensitivity scale the precision to be ~16 nm (at 0.2 s). The dynamic range is maintained at ~130 dB.

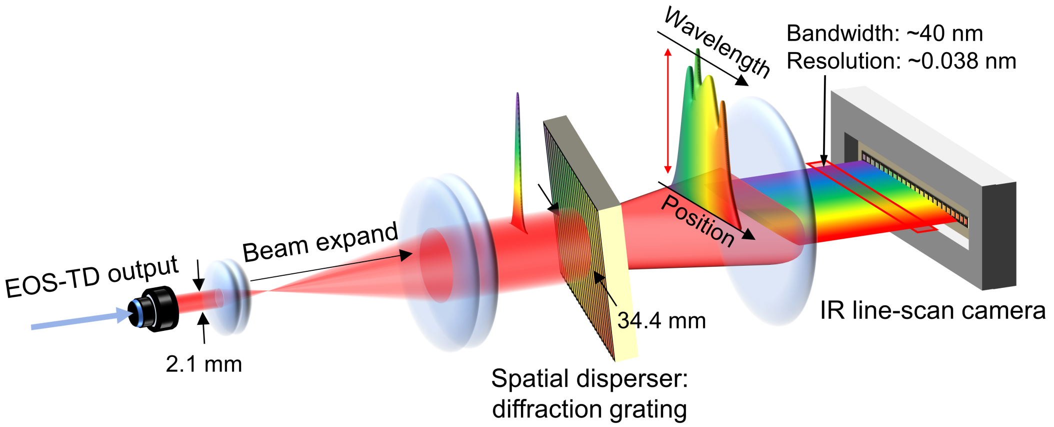

Figure S8 | Schematic diagram of spectral encoding and spectrum acquisition parts. The input collimated beam is expanded ~6 times to be ~12.9 mm in diameter. When incident to a 1,200 grooves mm^{-1} grating with an angle of 68 degrees, the beam size along the groove direction is ~34.4 mm, resulting in ~0.038 nm spectral resolution at 1,557 nm central wavelength. The spectrally dispersed beam is focused using a doublet lens. In the spectrum acquisition part, a f = 100 mm lens is used to focus the beam on the photodiode array (of line-scan camera) to be ~12.6 mm x ~30 µm in size. The camera's 1,024 photodiodes have 12.5 µm in pitch, matching the lateral resolution.

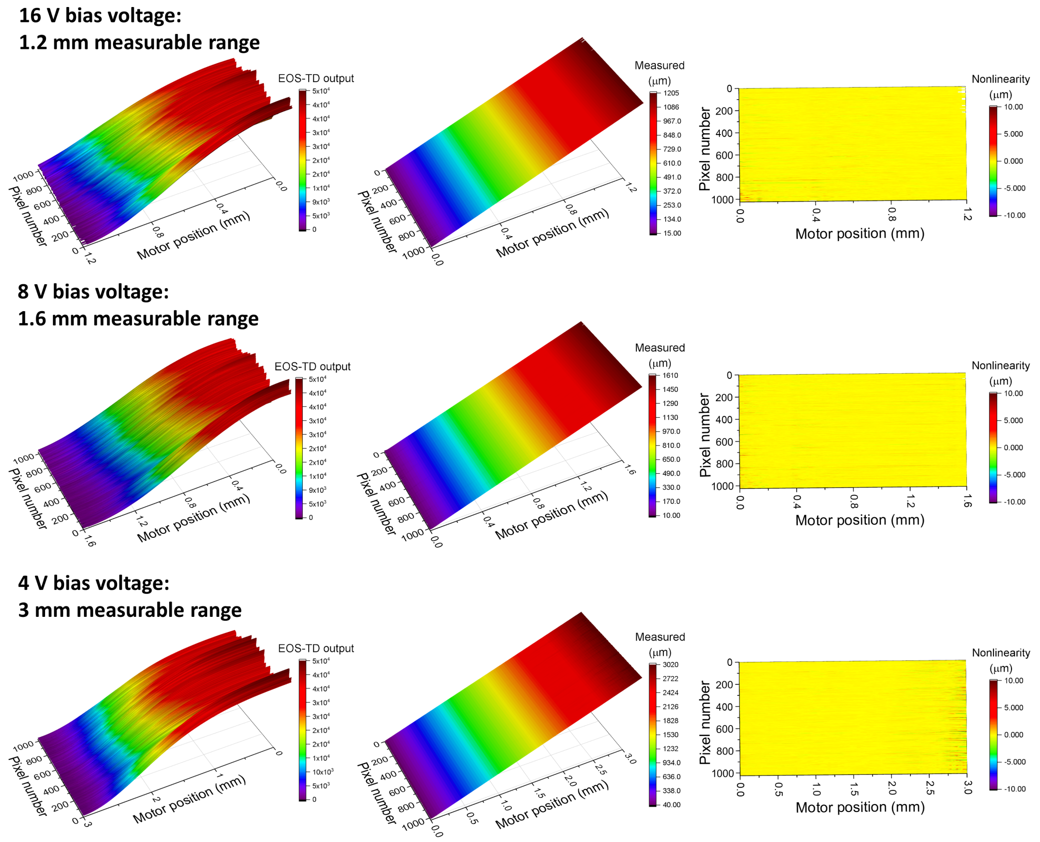

Figure S9 | Linearity measurements and measurable range variation according to the MUTC-photodiode bias voltages. (Left) Measured EOS-TD responses with respect to the motor position. (Middle) Calibrated EOS-TD responses. (Right) Remained nonlinearities. The measurable ranges at 16, 8, and 4 V bias voltage are ~1.2, ~1.6, and ~3 mm, respectively. The repeatability of the motor stage contributes to most of the nonlinearity, resulting in ~100 nm nonlinearity. At the ends of the measurable range, the nonlinearity increases due to the low slope of the electric waveform (where TOF detection sensitivity is lower).

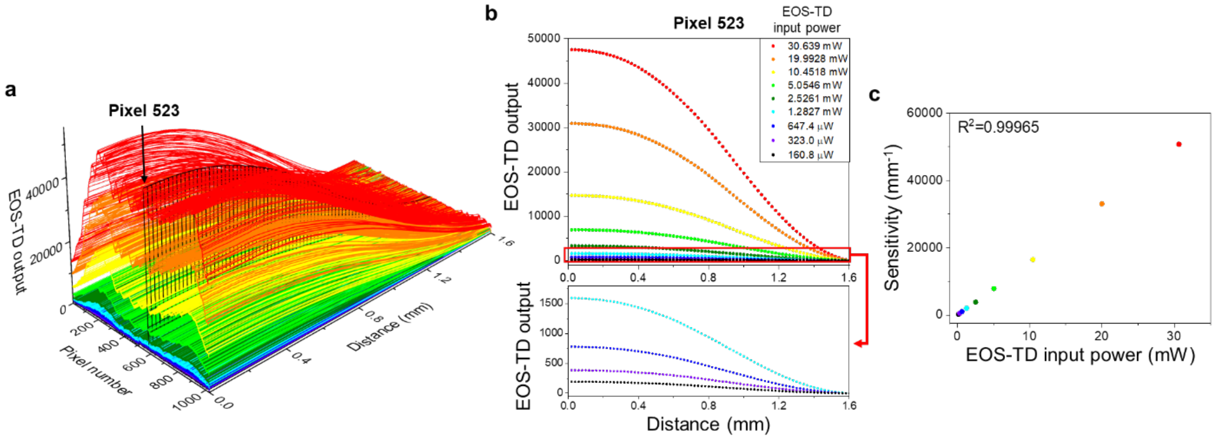

Figure S10 | Look-up table measurements with different input optical power. a, While reducing the input power, a precision motor stage (±100 nm repeatability) repeatedly scans the timing to measure look-up tables. The MUTC-photodiode is driven with 8 V bias voltage. **b,** The EOS-TD responses of a pixel (523rd). The enlarged graph of the region indicated by the red box is shown in the bottom panel. **c,** Sensitivity as a function of input power. The sensitivities show high linearity (R^2>0.999). The power-dependent EOS-TD response is derived by interpolating between the measured sensitivities.

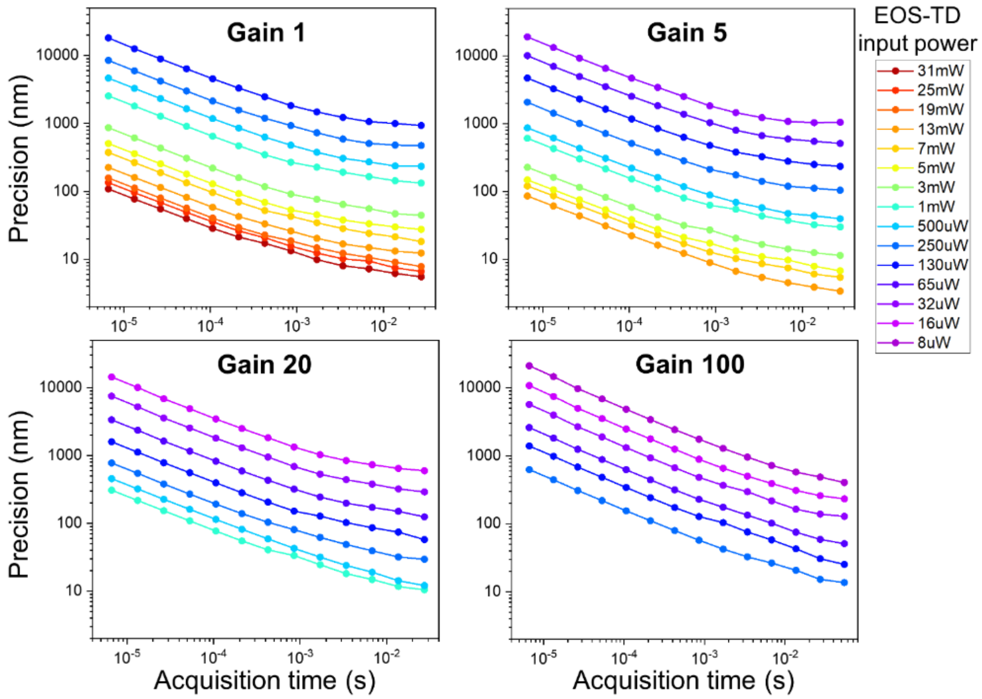

Figure S11 | Axial precision measurements for low input optical power. The camera exposure time is set to be 5 µs. When input optical power is low, the precision deterioration at fixed acquisition time can be prevented by increasing the camera electrical gain. As a result, with only 8 µW input power, ~400 nm precision can be acquired at 60 ms acquisition time. The precision at low input power can be further improved by increasing the exposure time.

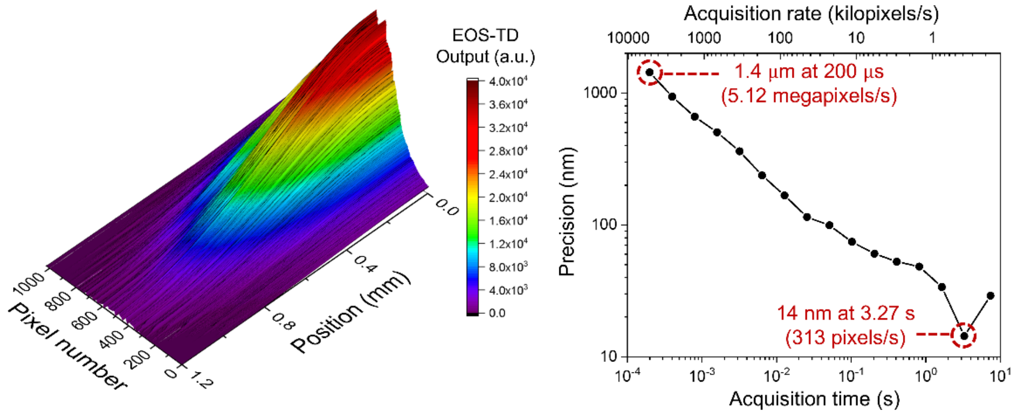

Figure S12 | Precision performance at ~1 µW input optical power. The input power is significantly decreased down to 1 µW, assigning ~200 pW equivalent power for each camera pixel. By increasing the camera exposure time and electrical gain to 200 µs and 100 times, respectively, all the 1,024 pixels could detect TOF variation, with best TOF measurement precision at 14 nm.

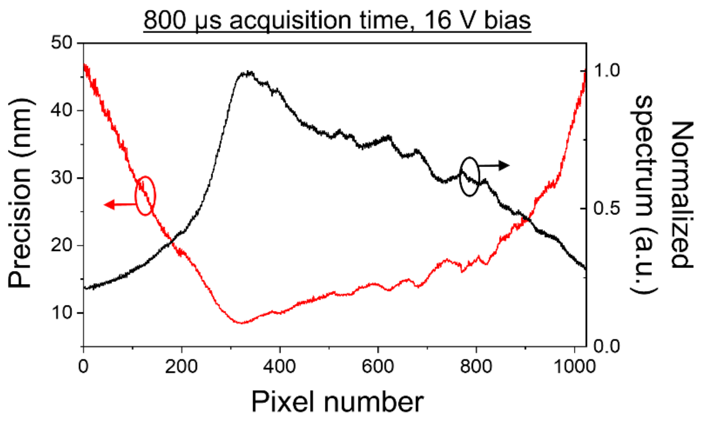

Figure S13 | TOF precision performance at each pixel position when sub-pulses' optical spectrum is non-uniform. TOF precision measurement at each pixel position at 800 μs acquisition time (16 V bias, unsaturated camera). The spectral intensities at both boundaries are ~80% less than the maximum intensity (pixel 330). As a result, the precision performances at pixel 1 and pixel 1024 are 47 nm and 45 nm, respectively, while the precision at pixel 330 is 8.42 nm.

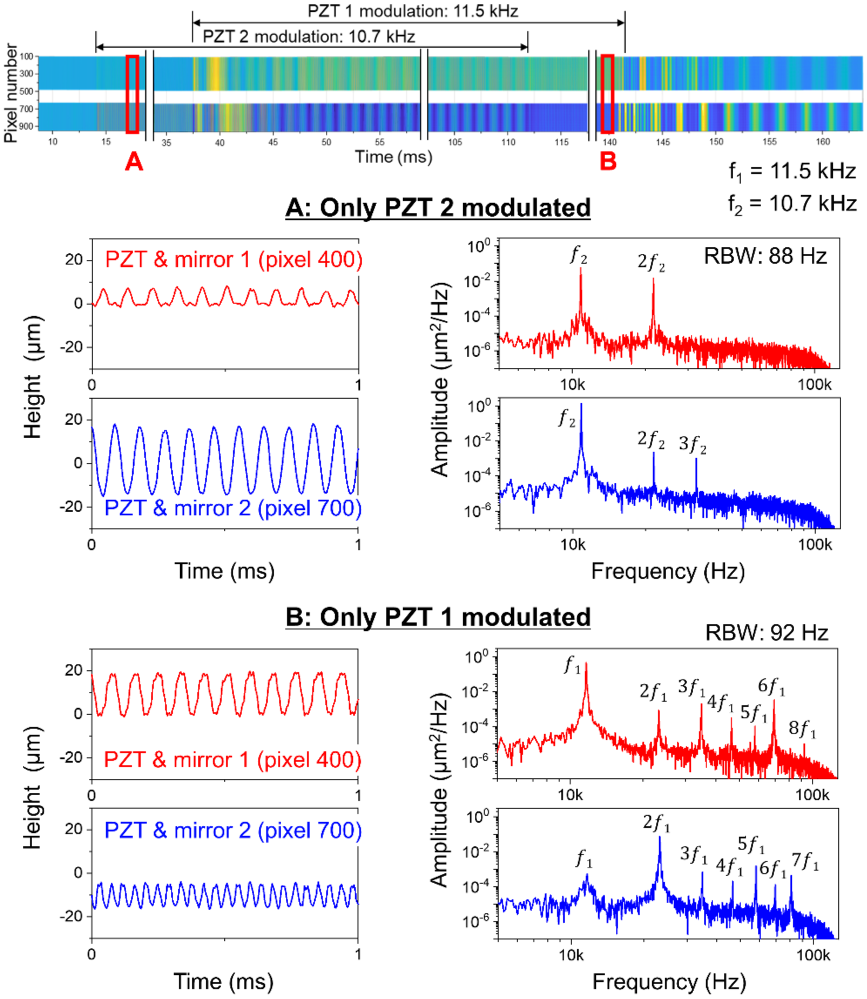

Figure S14 | Fourier transformed spectra of PZT interaction measurements. Time traces of pixel 400 and pixel 700 at region A (when only PZT 2 is modulated with 10.7 kHz (f_2) frequency) and region B (when only PZT 1 is modulated with 11.5 kHz (f_1) frequency) are Fourier transformed. At region A, in PZT 2-mounted mirror, the resonance mode (f_2) is excited with highest amplitude and high-harmonic components up to 3rd harmonic frequency are also excited, and the first two harmonic modes are coupled to PZT 1-mounted mirror. The coupled two modes show similar amplitudes, resulting in non-single-tone fluctuation in the time-domain. At region B, while the resonance frequency (f_1) has highest amplitude, high-harmonic components up to 8th harmonic frequency are excited in PZT 1-mounted mirror. In PZT 2-mounted mirror, the coupled harmonic components up to 7th harmonic frequency are observed in the Fourier transformed spectrum, and the 2nd harmonic component ($2f_1$) appears with the highest amplitude. As a result, while PZT 1 is modulated with 11.5 kHz, the affected PZT 2-mounted mirror mainly fluctuates with 23.0 kHz frequency.

Since the two PZT-mounted mirrors have similar resonance frequencies and resonance modes have broad linewidths, each PZT-mounted mirror can be excited with the resonance frequency (and harmonic frequencies) of each other. The coupling coefficients between PZT 1's harmonic components (f_1, $2f_1$, $3f_1$, …) and PZT 2's harmonic components (f_2, $2f_2$, $3f_2$, …) can be all different depending on their linewidths, geometries, driving force, and other factors. Additionally, because the PZT-mounted mirrors have different geometry and are not operated in vacuum, the mode coupling may be produced with much more complexity.

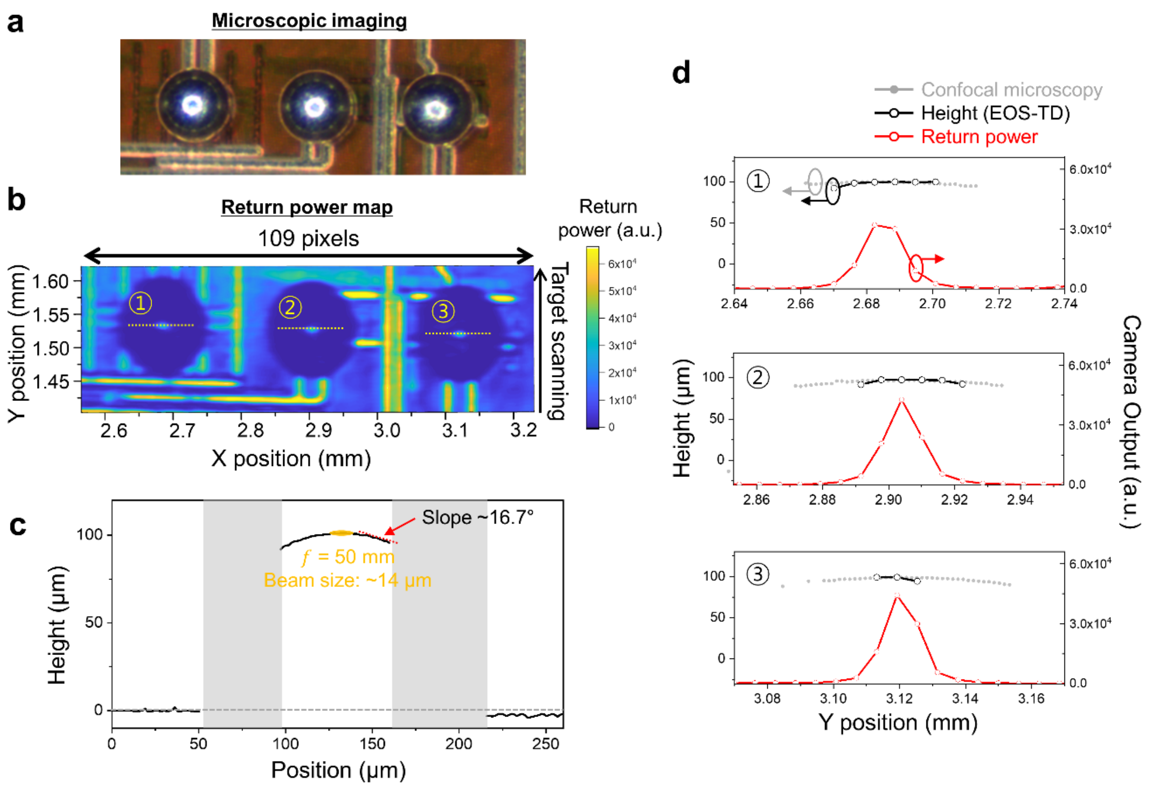

Figure S15 l Round-shape surface measurement result. **a**, Microscopic image of micro-bumps. The bumps have ~125 μm diameter, ~100 μm height, and spaced by ~250 μm in pitch. **b**, Returned power map (measured by line camera) after illuminating with line beam and scanning the bumps in Y-direction. The dark blue region is where there is no power return. A f = 50 mm lens is used for longer Rayleigh range (>100 μm) and calculated NA of the system is 0.25. **c**, Cross section of a bump measured by a confocal microscopy and slopes. The grey region is where confocal microscopy fails to measure height. **d**, Return power and measured TOF for the three bumps in **b**.

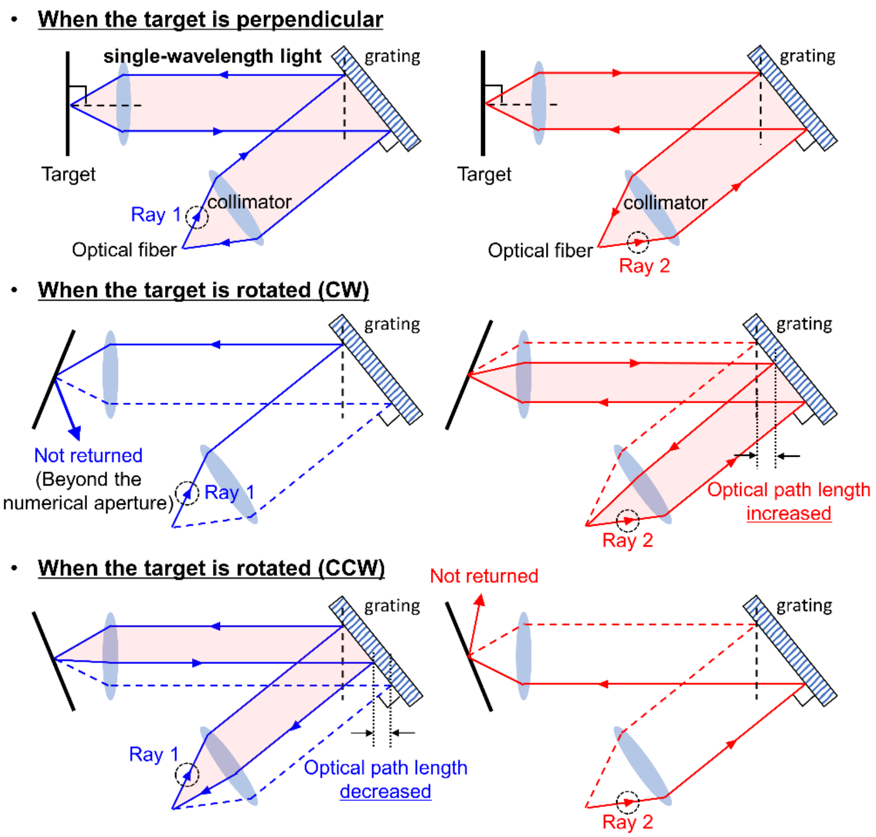

Figure S16 l Schematic diagram of optical path length variation when target surface rotates or has a slope. To simplify the situation, the collimated single-wavelength beam from optical fiber and collimator is incident to the grating in perpendicular, and after diffraction, the collimated beam is focused on to the target surface. Ray 1 and Ray 2 indicate the upper-end and lower-end rays departing from optical fiber, respectively. CW, clockwise; CCW, counter-clockwise.

	Maximum sub-pulse power	Minimum sub-pulse power
**(Condition 1) Gain 1, exposure time 2.25 μs**	70 μW	52 nW
**(Condition 2) Gain 100, exposure time 2.25 μs**	554 nW	2.3 nW
**(Condition 3) Gain 100, exposure time 500 μs**	2.63 nW	0.82 pW

Table S1 l The maximum and the minimum sub-pulse's average power input to the EOS-TD with various camera operating conditions.

Supplementary References

S1. Kato, T., Uchida, M., Tanaka, Y. & Minoshima, K. High-resolution 3D imaging method using chirped optical frequency combs based on convolution analysis of the spectral interference fringe. *OSA Contin.* **3**, 20 (2020).

S2. Mizuno, T. *et al.* Computationally image-corrected dual-comb microscopy with a free-running single-cavity dual-comb fiber laser. *Opt. Express* **29**, 5018 (2021).

S3. Joo, W.-D. *et al.* Femtosecond laser pulses for fast 3-D surface profilometry of microelectronic step-structures. *Opt. Express* **21**, 15323–15334 (2013).

S4. Huang, D. *et al.* 400 MHz ultrafast optical coherence tomography. *Opt. Lett.* **45**, 6675 (2020).

S5. Wang, Z. *et al.* Cubic meter volume optical coherence tomography. *Optica* **3**, 1496 (2016).
